# Soil consolidation acoustic experiment and pore pressure prediction model establishment—taking the Yingqiong Basin as an example

**DOI:** 10.1038/s41598-023-29078-x

**Published:** 2023-02-02

**Authors:** Zhongying Han, Bo Sun, Yuanfang Cheng, Chuanliang Yan

**Affiliations:** 1 School of Petroleum Engineering, China University of Petroleum (East China), Qingdao, 266580 China; 2 Key Laboratory of Unconventional Oil & Gas development (China University of Petroleum (East China)), Ministry of Education, Qingdao, 266580 China

**Keywords:** Crude oil, Natural gas

## Abstract

To establish a pore pressure prediction model suitable for the Yingqiong Basin in the South China Sea. A new laboratory test method was designed to simulate the core consolidation process, and an acoustic experiment for soil consolidation was carried out to analyze various parameters and establish an abnormal pore pressure prediction model suitable for the target block. First, the cause of abnormally high pressure in the Yingqiong Basin is analyzed and identified, and a simulation experiment of stratum loading and unloading is performed. The soil consolidation, experimental equipment and experimental plan are designed. Several sets of experiments were carried out, the changes in various parameters during the experiment were analyzed and summarized, the pore pressure prediction model suitable for this area was standardized and established, and application verification and evaluation were carried out in some wells in this area. The results show that the prediction model is accurate and applicable, and the pore pressure can be predicted by the soil consolidation experiment method that the prediction accuracy is slightly better than the traditional prediction model. In this study, a feasible soil consolidation acoustic experiment method can be used to establish a rock mechanics experiment and a pore pressure prediction model without strict core rock. The experiments have proved the feasibility of this method and obtained two prediction models, including loading mechanism and unloading mechanism prediction models.

## Introduction

According to the evaluation of oil and gas resources by the Ministry of Natural Resources, the Yinggehai and Qiongdongnan basins account for more than 80% of the offshore resources^1^, with outstanding characteristics of overflow and lost circulation^2^. The Ledong and Yacheng gas fields are important oil and gas resource blocks in the western South China Sea oilfields, containing abundant oil and gas resources, but the geological conditions are complex, given the channel sand development^3^. High-temperature and high-pressure wells have a narrow density window, and complex situations such as overflow and leakage which occur frequently. Take nine high-temperature and high-pressure exploratory wells in a block in the Yingqiong Basin as an example, these wells with complex well control conditions all have overflow, and six of them have lost circulation; the operating density window is less than 0.11, and some wells even have negative windows. The drilling was forced to stop while drilling to seal three wells.

There are various causes of abnormally high pressure, including geological, physical, geochemical and dynamic factors. For abnormally high pressure in a certain block, the cause of such abnormally high pressure is generally dominated by a certain factor, and supplemented by other factors^4^. Although there are many mechanisms for the formation of abnormally high pressure^5,6^, hydrocarbon generation and undercompaction have always been the most common mechanisms for abnormally high pressure^7^. Hydrocarbon generation belongs to the abnormally high pressure caused by the change in pore fluid volume, and undercompaction belongs to the abnormally high pressure caused by the change in rock pore volume.

There are different calculation models for abnormally high pressures formed by different mechanisms, such as the Eaton method^8^ and the equivalent depth method^9^ for the loading mechanism (undercompaction) and the Bowers method^10,11^ for the unloading mechanism. Some progress has been made in the field of pressure testing research at home and abroad, but the above research results are not suitable for the formation pressure prediction of high-temperature and high-pressure wells in the Yingqiong Basin, and the pressure calculation method has large errors. It is urgent to carry out research on the formation pressure calculation model of the Yingqiong Basin.

The pore pressure prediction model proposed in the past was based on well logging data after inductive analysis and modeling. The model was obtained based on regional data, and the regional applicability was highly dependent, and it did not pass strict rock mechanics experimental verification and research. Performing rock mechanics experiments by complete coring of underground rocks is not only difficult to coring, but the in situ stress state of the rock mass after extraction also changed. Therefore, there are large errors in the experiment of this method. This study is based on laboratory experiments using South China Sea strata cuttings. According to the formation mechanism of different compression mechanisms, the core is homemade, and indoor artificial core samples are used for soil consolidation acoustic experimental research and analysis. The research variables are controlled, and the parameter relationship is summarized. Fitting and deriving the pore pressure prediction model is established through carrying out pore pressure prediction and modeling by the method of soil consolidation experiments, which solves the shortcomings of the above methods, and the prediction accuracy is slightly better than that of other traditional prediction models. The soil consolidation experiment method used in this study allows the establishment of rock mechanics experiments and pore pressure prediction models without rigorous underground coring but also makes up for the lack of experimental basis for previous empirical models.

In this study, through the method of indoor soil consolidation acoustic experiments, using the acoustic wave and density response law of the mudstone consolidation process, research on the formation mechanism of abnormally high pressure in the Ledong and Yacheng blocks is carried out, and the measurement of the pressure formation process of the loading and unloading mechanism is studied. Well data response law, help establish formation pressure calculation model suitable for the target block, which not only provides experimental science for pore pressure prediction under different compression mechanisms but also provides technical support for effective and precise managed pressure drilling in abnormally high-pressure blocks and promotes the safe and efficient development of blocks in the Yingqiong Basin.

## Material and method

### Identification of the volume characteristic parameters

**Figure 1:**
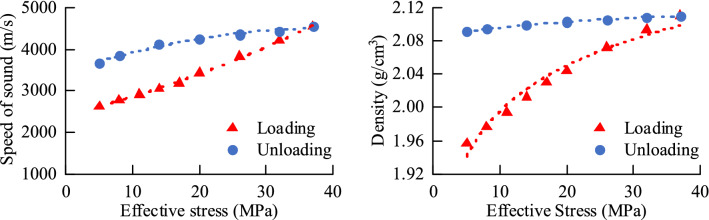
Relationship between sound wave velocity or density and effective stress.

According to the experimental data analysis of mudstone by Bowers^10,11^ and Tosaya^12^, the formation mechanism of abnormally high pressure can be identified by using the sonic-density intersection map^13,14^. During the loading (undercompaction) process, the wave velocity and the physical property parameters of the density and density both increase with the effective stress; during the unloading process (hydrothermal pressurization, hydrocarbon generation, fluid charging), as the effective stress decreases, the wave velocity also decreases significantly, while the density data remain basically unchanged. Acoustic velocity and resistivity, density and porosity are conduction characteristics and volume characteristics, respectively. When the rock is subjected to unloading, the pore volume increases, the effective stress decreases, and the conduction characteristics, such as acoustic velocity, are more sensitive to the change in the unloading mechanism, so there will be obvious changes, but the volume characteristic parameters, such as density and porosity, are basically unchanged^15^. As shown in Fig. 1, under the action of the loading mechanism, the effective stress of the rock increases, and the sound wave velocity and density also increase significantly; under the action of the rock unloading mechanism, the effective stress decreases, and the sound wave velocity shows a decrease due to the effective stress. The trend gradually decreases, but the density is basically unchanged at this time. Therefore, the type of abnormal high-pressure formation mechanism can be analyzed according to the different response characteristics of acoustic wave velocity and density when the formation is loaded or unloaded^16,17^.

**Figure 2:**
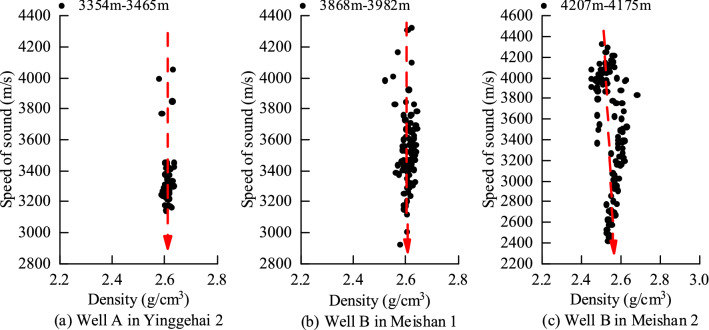
Abnormal pressure identification of some wells in the Ledong block.

Well A in the Ledong block of the Yingqiong Basin is located at depths of 3354–3465 m in the second member of the Yinggehai Formation. The acoustic characteristics and density in the logging are analyzed. The data show that the density is basically unchanged, and the acoustic velocity decreases, as shown in Fig. 2a, and it conforms to the regular characteristics of the unloading mechanism. Similarly, the analysis of the 3868–3982 m section of the first member of the Meishan Formation and the 4027–4175 m section of the second member of the Meishan Formation in Well B, as shown in Fig. 2b,c below, both show obvious unloading mechanisms.

**Figure 3:**
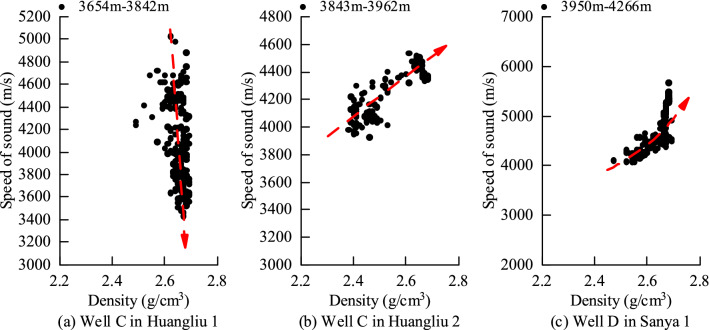
Abnormal pressure identification of some wells in the Yacheng block.

Well C in the Yacheng block of the Yingqiong Basin is located at depths of 3654–3842 m and is located in the first member of the Huangliu Formation. The acoustic characteristics and density in the logging are analyzed. The data show that the density is basically unchanged, and the acoustic velocity decreases, as shown in Fig. 3a below. As shown, it conforms to the regular characteristics of the unloading mechanism.

Well C in the Yacheng block of the Yingqiong Basin is located at depths of 3843–3962 m and is located in the second member of the Huangliu Formation. The acoustic characteristics and density in the logging are analyzed. The data show that the density and acoustic velocity increase at the same time, as shown in Fig. 3b below. It is shown that it conforms to the regular characteristics of the loading mechanism. Similarly, the analysis of the 3950–4266 m section of the first member of the Sanya Formation in Well D, as shown in Fig. 3c below, also shows an obvious loading mechanism.

Therefore, the abnormally high pressures in the Ledong and Yacheng areas located in the Yingqiong Basin are similar in origin. There are mainly two obvious pressure-forming mechanisms, namely, the loading mechanism and the unloading mechanism.

### Experimental materials and equipment

Based on the effective stress theory, the experimental simulation is carried out to conduct the response experiment of acoustic velocity and density under abnormally high pressure.

In this study, both undercompaction and normal compaction belong to the loading process, and it is considered that the same effective stress produces the same porosity, but the loading rate of undercompaction is faster, resulting in abnormally high pressure. In this study, the process in which the effective stress decreases (the vertical stress remains unchanged and the pore pressure increases) is the unloading process. The main experimental variables in the experimental process are acoustic velocity, density, effective stress, pore pressure, and vertical pressure.

**Figure 4:**
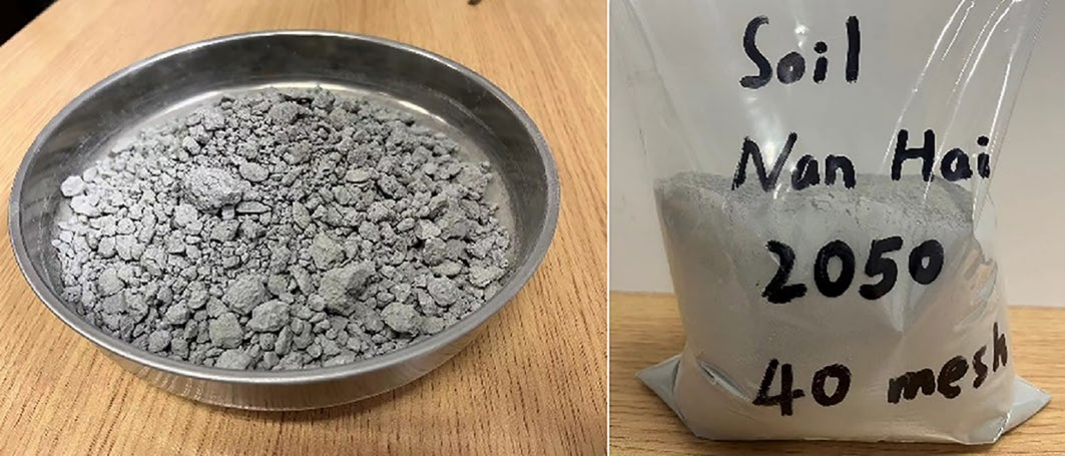
Formation cuttings and soil.

In this method, real formation cuttings in the Yingqiong Basin area were selected. The approximate location for obtaining the cuttings is 108.6° E and 17.7° N, and the depth is 2050 m. Before the experiment, the rock cuttings were crushed by a 40-mesh crusher, and the soil was formed. The experimental rock cuttings are shown in Fig. 4.

**Figure 5:**
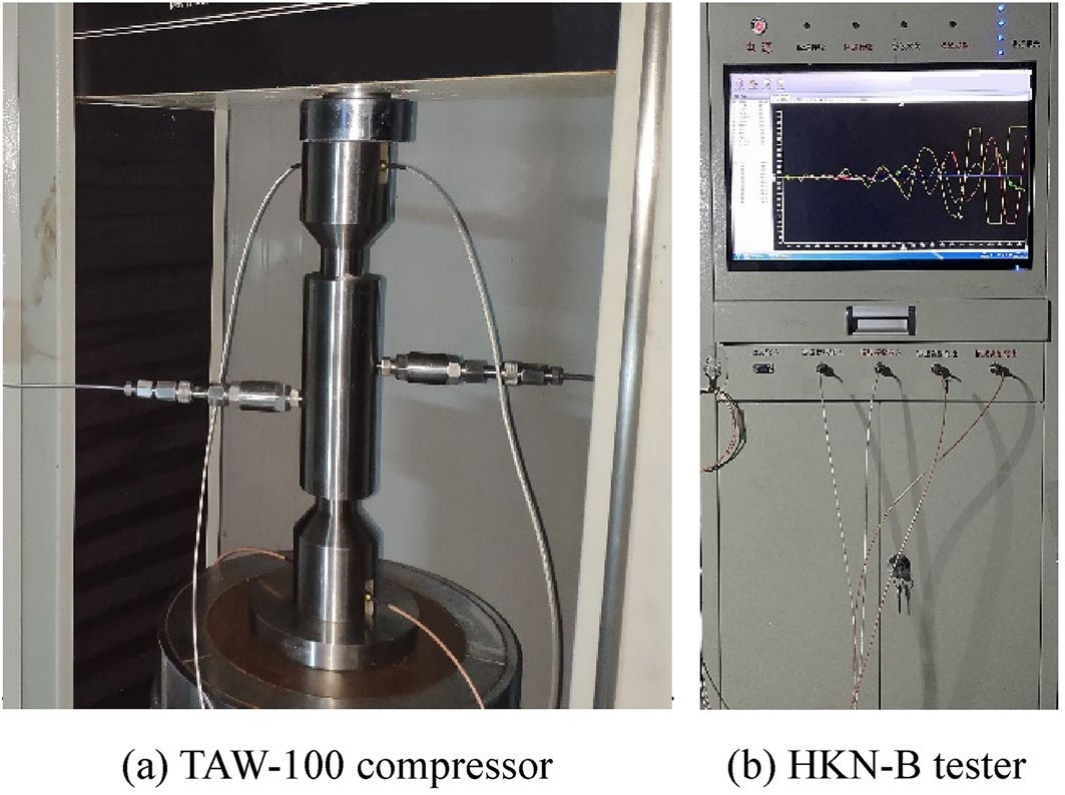
Equipment.

The equipment for this experiment includes two carriers, a pressure cylinder, and an acoustic wave sensor probe. Experimental auxiliary equipment includes a servo control compressor, acoustic testing system, computer, high-pressure pipeline, acoustic wave test line, sealing ring, etc. Among them: the model of the servo-controlled compressor is TAW-100, as shown in Fig. 5a, which can record displacement changes with high precision in real-time, and the precision of pressure and displacement is 0.1%. The model of the acoustic testing system is HKN-B, as shown in Fig. 5b, the time readout accuracy is 0.05 microseconds; this experiment adopts the direct penetration method to collect the sound wave velocity, to avoid the influence of refracted waves, scattered waves, etc., the experimental equipment has taken corresponding measures to weaken the influence of refracted waves and scattered waves. For example, the method of notching the upper and lower carrier cylinders reduces the influence of scattered waves and surface waves; at the same time, to avoid the systematic error of the acoustic wave test, the acoustic wave time difference between the two acoustic wave sensors and the acoustic wave test instrument is directly measured, and they are eliminated. Eliminating the self-delay between them makes the experiment more rigorous.

**Figure 6:**
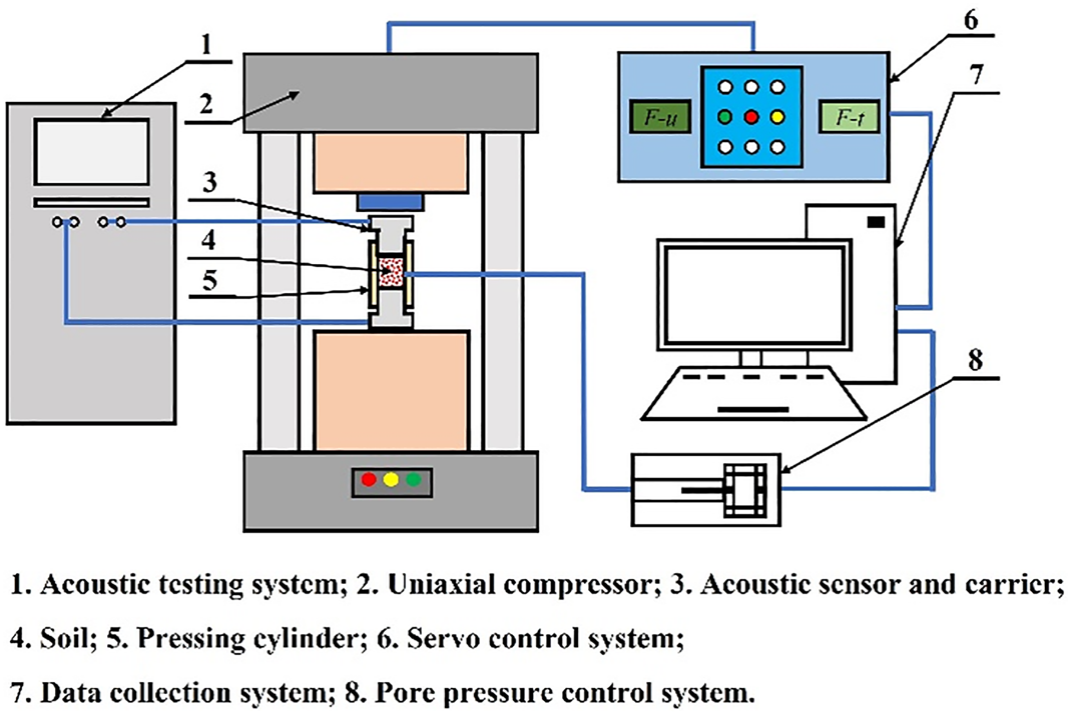
The schematic diagram of the experimental system.

During the soil consolidation equipment, a carrier should be installed in the pressure cylinder, and the pressure cylinder should be filled with soil required for the experiment. Another carrier should be installed in the pressure cylinder, and the two acoustic wave sensors should be installed in the carriers. Soil consolidation equipment is shown in the middle of Fig. 5a. The soil consolidation equipment is placed under the high-precision servo-controlled compressor, the acoustic testing system and the acoustic wave sensor are connected by the acoustic wave line, the pressure cylinder wall hole and the pore pressure control system are connected by the high-pressure pipeline. The schematic diagram of the experimental system is shown in Fig. 6.

### Experiment scheme design

#### Experiment group 1: simulation of loading experiment

Initial experimental parameters: pore pressure 5 MPa, vertical pressure 10 MPa.

*Normal compaction* The pore pressure and vertical pressure are gradually loaded at the same rate after a period of time (60 min) from the initial experimental values. The pore pressure and vertical pressure increase, and the effective stress increases, but the pore pressure equivalent density remains unchanged. Example: the pore pressure is loaded to 10 MPa, while the vertical pressure is loaded to 20 MPa. That is, the effective stress increases, and the pore pressure equivalent density does not change.

*Undercompaction* according to the same effective stress to produce the same degree of compaction, according to the normal compaction curve, obtain the sound wave velocity and density under different equivalent pore pressures. During the experiment, after the core displacement became stable, the experimental parameters, such as acoustic velocity and displacement, were measured.

Change the loading rate and perform multiple experiments in this scheme, but the variation law of real-time effective stress and pore pressure equivalent density during normal compaction or undercompression during core loading should be guaranteed. The parameters of each experimental point in experimental Group 1 are shown in Table 1 below.

**Table 1:** Parameters of each experimental point in the loading experiment.

Load Test	Pore Pressure MPa	Effective Stress MPa	Vertical Stress MPa
Starting	5	5	10
Load Time		60 min	
Hold Time		24 h	
Point 1	6	6	12
Point 2	8	8	16
Point 3	10	10	20
Point 4	12	12	24
Point 5	14	14	28
Point 6	16	16	32
Point 7	18	18	36
Point 8	20	20	40
Point 9	22	22	44
Point 10	24	24	48
Point 11	26	26	52
Point 12	28	28	56
Point 13	30	30	60

#### Experiment group 2: simulated of unloading experiment

Initial experimental parameters: Example: pore pressure 5 MPa, vertical pressure 10 MPa.

The pore pressure and vertical pressure are from the initial experimental values, and after a period of time (for example: 60 min), they are gradually loaded to a certain value by constant rate and normal compaction (for example, the pore pressure is loaded to 30 MPa, and the vertical pressure is loaded to 60 MPa). Keep for a period of time (24 h).

Keeping the vertical pressure constant, the increase in pore pressure reduces the effective stress. Example: the vertical pressure remains unchanged at 60 MPa, and the pore pressure increases from 30 to 40 MPa after a period of time (60 min). That is, the effective stress decreases, and the pore pressure equivalent density increases. During the experiment, after the core displacement became stable, the experimental parameters, such as acoustic velocity and displacement, were measured.

It is possible to change the unloading rate and repeat this scheme several times, but it should be ensured that the core unloading effect and the effective stress increase and the pore pressure equivalent density increases. The parameters of each experimental point in experimental Group 2 are shown in Table 2 below.

**Table 2:** Parameters of each experimental point in the unloading experiment.

Unload Test	Pore Pressure MPa	Vertical Stress MPa	Effective Stress MPa
Starting	30	60	30
Test Time		60 min	
Hold Time		24 h	
Point 1	32	60	28
Point 2	34	60	26
Point 3	36	60	24
Point 4	38	60	22
Point 5	40	60	20
Point 6	42	60	18
Point 7	44	60	16
Point 8	46	60	14
Point 9	48	60	12
Point 10	50	60	10
Point 11	52	60	8
Point 12	54	60	6
Point 13	55	60	5

## Results and models

### Results of loading experiments

**Figure 7:**
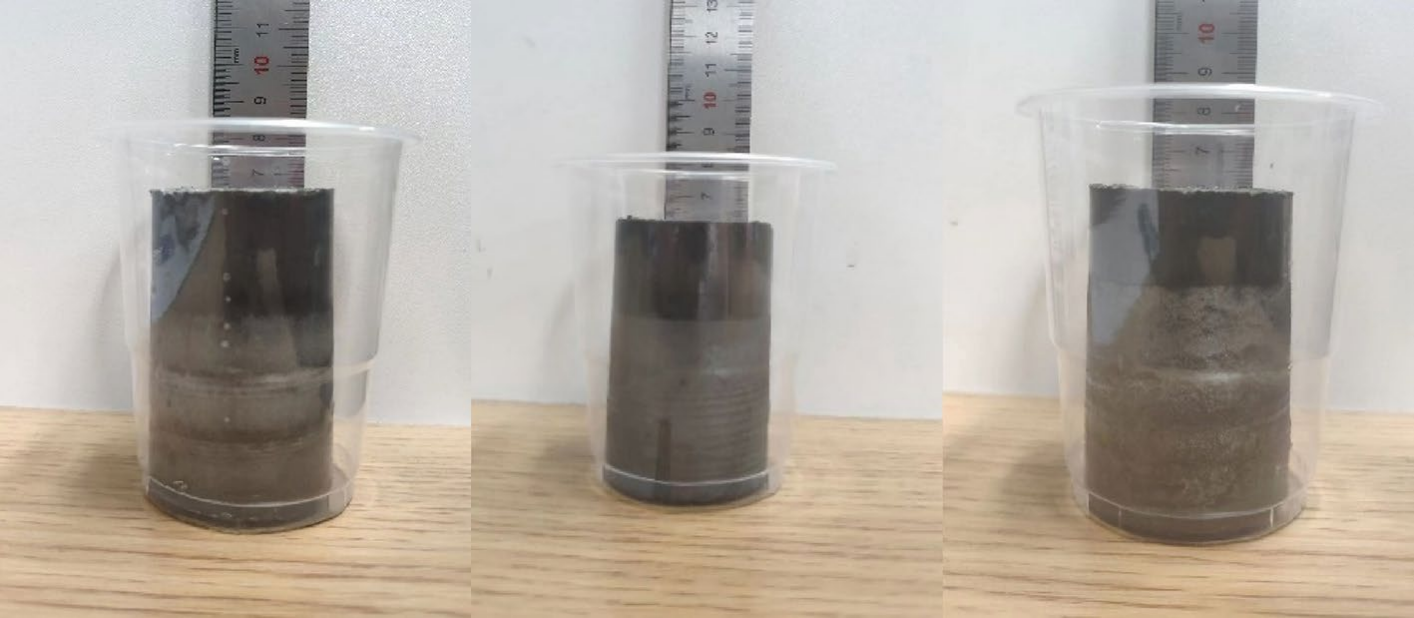
Rock after soil consolidation.

The loading experiment in this study carried out multiple sets of experiments (After the test, the soil consolidated core is shown in Fig. 7), and only one set of experimental results is shown here for analysis and description. Sample parameters of experimental Group 1: lithology: mudstone; sampling depth: 2050 m; mud content: 0.346.

Loading experiment: simulate the normal compaction process, increase the axial pressure and pore pressure, and gradually increase the effective stress. After the consolidation becomes stable, the acoustic velocity, density and other parameters of each effective stress point are measured. The effective stress of experimental Group 1 gradually increased from 5 to 30 MPa.

Normal compaction: the pore pressure is gradually increased from 5 to 30 MPa, and the vertical pressure is correspondingly increased from 10 to 60 MPa so that the effective stress is gradually increased from 5 to 30 MPa.

**Figure 8:**
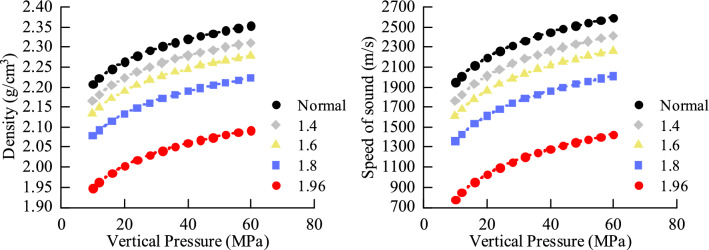
Laws of acoustic wave velocity and density under different equivalent pore pressures.

Undercompaction: According to the same effective stress to produce the same degree of compaction, according to the normal compaction curve, the sound wave velocity and density under different equivalent pore pressures are obtained, and a graph is drawn, as shown in Fig. 8 below. The density increases with increasing effective stress, and the acoustic velocity increases with increasing effective stress.

### Results of unloading experiments

**Figure 9:**
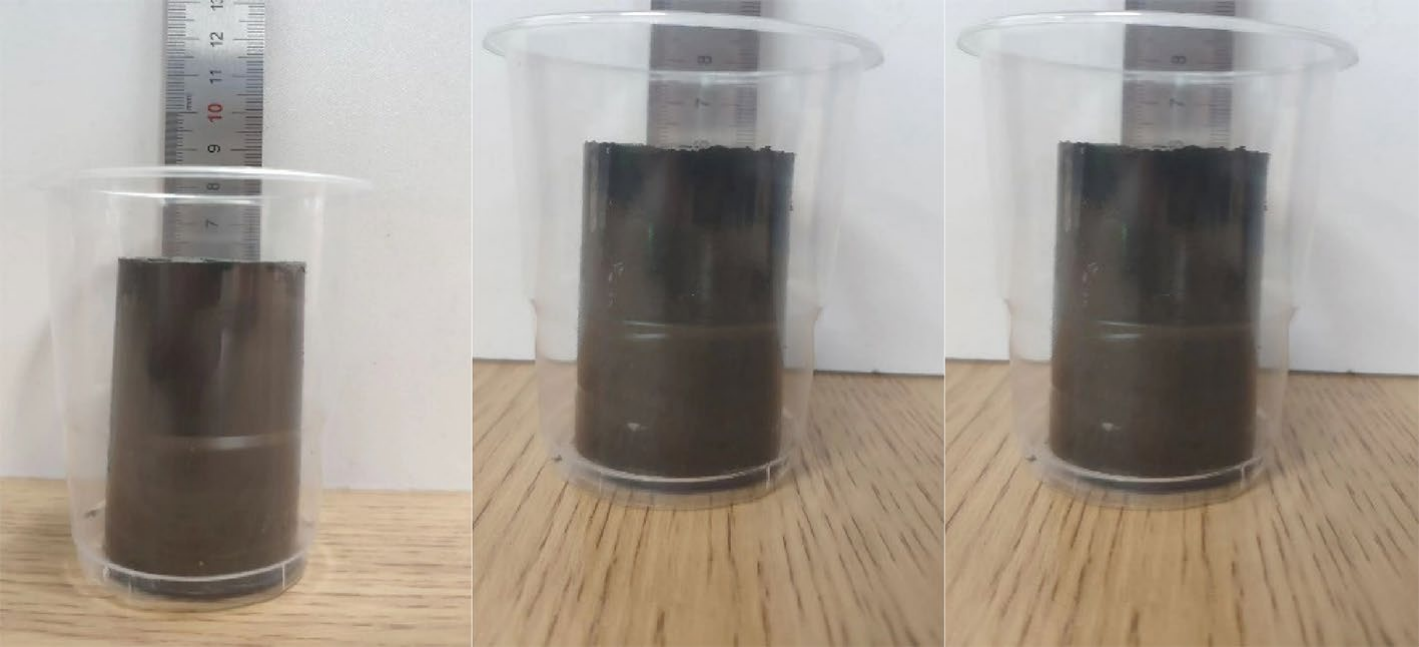
Rock after soil consolidation.

The unloading experiment in this study also carried out multiple sets of experiments (After the test, the soil consolidated core is shown in Fig. 9), and only one set of experimental results is shown here for analysis and description.

**Figure 10:**
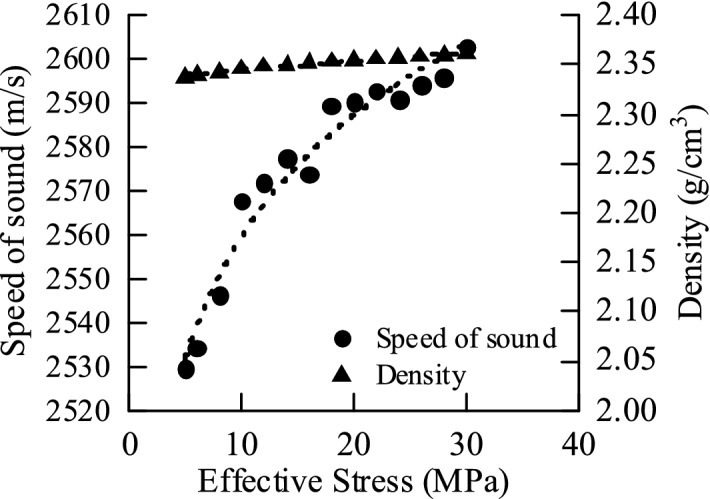
Results of Unloading experiments.

Simulate the unloading process. After the normal compaction becomes stable, the axial pressure remains unchanged, and the pore pressure is increased to gradually reduce the effective stress. After the experiment is stabilized, parameters such as the sound wave velocity and density of each effective stress point are measured, and the effective stress ranges from 30 MPa. It is reduced to 5 MPa, and the starting point of unloading is 30 MPa. The data of each experimental result are shown in Fig. 10 below. The density remains basically unchanged as the effective stress decreases, and the acoustic velocity decreases as the effective stress decreases.

### Models of pore pressure prediction

#### Model of loading mechanism

Modeling process:

1. Normalized sound wave velocity (dimensionless):
  1 $$ V^{*} = \frac{{V_{0} }}{V} $$
  ${V}_{0}$: Ultrasonic velocity of Normal Compaction.$V$: Ultrasonic velocity of Measure.
2. According to the sound wave velocity maps under different equivalent pore pressures, the standardized sound wave velocity V* matrix and the loading effective stress matrix are established. The cross plot of V* and effective stress matrix data is shown in Fig. 11 below. Determine the fitting type as Eq. (2), fit and solve coefficient matrices a, b.
  2 $$ \sigma_{e} = a \cdot V*^{b} $$
  ${\sigma }_{e}$: Effective stress. $a, b$: Coefficient.

**Figure 11:**
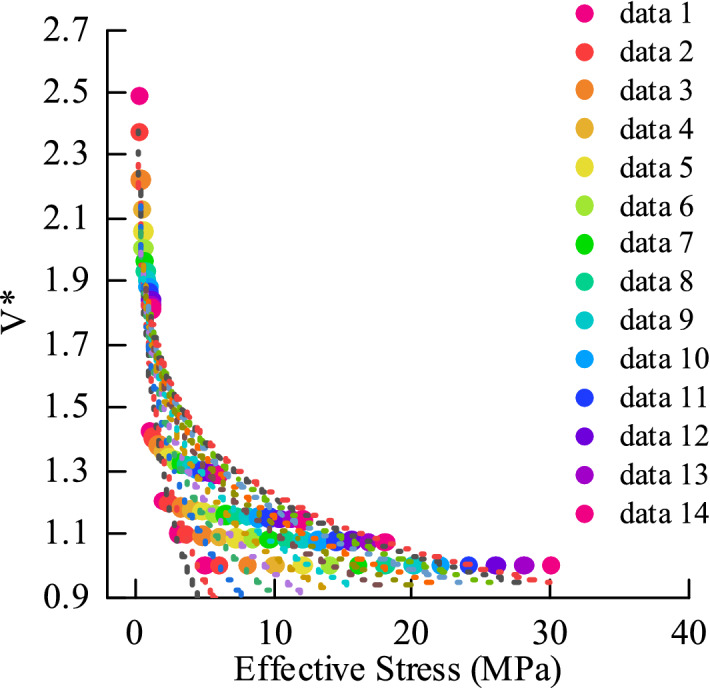
V* and effective stress matrix data.
3. Establish the relationship between the a and b coefficient matrices and overburden pressure, fit and parameterize a and b (based on sample 1)
  3 $$ \begin{gathered} a = 0.4969\sigma_{v} - 0.0158 \hfill \\ b = - 0.998\ln (\sigma_{v} ) - 2.4664 \hfill \\ \end{gathered} $$
  $${\sigma }_{v}$$: Overburden Pressure.
4. For sample 2, model the same as the above modeling process, and obtain the parameters a and b of sample 2:
  4 $$ \begin{gathered} a = 0.4979\sigma_{v} - 0.0087 \hfill \\ b = - 0.999\ln (\sigma_{v} ) - 5.707 \hfill \\ \end{gathered} $$
5. Combining the experimental results of other groups, the loading model is obtained:
  5 $$ \begin{gathered} \sigma_{e} = 0.5\sigma_{v} \cdot \left( {\frac{{{\text{V}}_{0} }}{{\text{V}}}} \right)^{{ - \ln (\sigma_{v} ){ - }d}} \hfill \\ d = 0.5 - 3 \hfill \\ \end{gathered} $$

#### Model of unloading mechanism

1. Standardized acoustic velocity (dimensionless), standardized unloading starting stress (dimensionless):
  6 $$ V* = \frac{{V_{q} }}{{V_{{}} }} \quad \sigma_{e*} = \frac{{\sigma_{eq} }}{{\sigma_{e} }} $$
  ${V}_{q}$: Ultrasonic velocity at the starting point.$V$: Ultrasonic velocity of Measure.${\sigma }_{eq}$: Effective stress at the starting point.
2. Establish the $\sigma_{e*}$ matrix and V* matrix. The intersection of the V* and $\sigma_{e*}$ matrix data is shown in Fig. 12 below. Determine the fitting type as Eq. (7), and fit and solve coefficient matrices a and b (based on sample 1):
  7 $$ \sigma_{{e{*}}} = aV*^{b} $$

**Figure 12:**
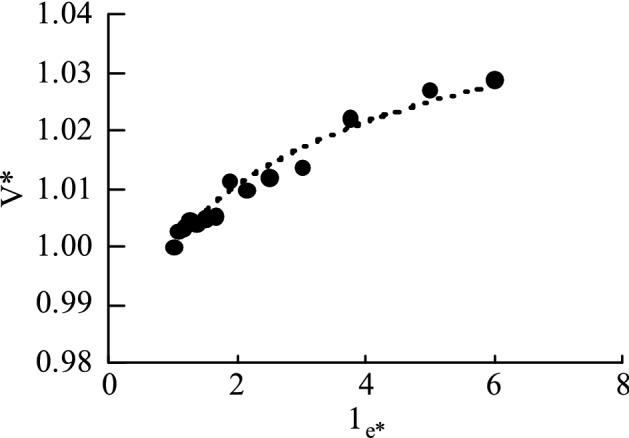
V* and σe ∗ matrix data.
3. Combining the experimental results of other groups, the unloading model is obtained:
  8 $$ \begin{gathered} \sigma_{e} = \frac{{\sigma_{eq} }}{{a\left( {\frac{{V_{q} }}{V}} \right)^{b} }} \hfill \\ a = {1}{\text{.4, }}b = - {1}{\text{.34}} \hfill \\ \end{gathered} $$

### Application and evaluation of the model

**Figure 13:**
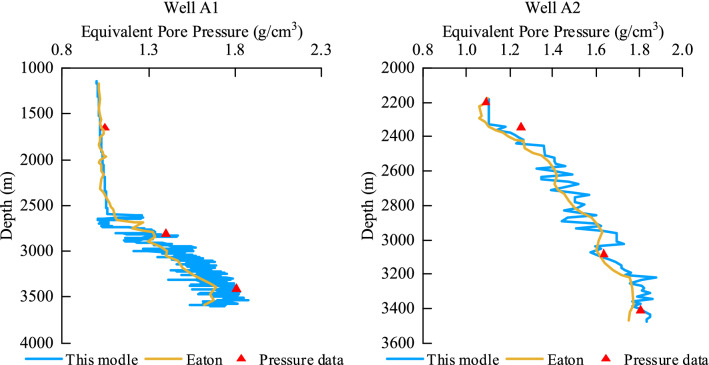
Comparison of loading modeling prediction results.

For Well A1, according to the logging data, the loading model is suitable for the well interval from 1000 to 3500 m. The pore pressure prediction (d = 0.5) is carried out according to the loading model in this study. The prediction results are shown in Well A1 in Fig. 13 below. The calculation results of the commonly used Eaton model are plotted in the figure. The calculation results of the prediction model in this study are compared with the Eaton model. According to the pressure measurement data, the total accuracy error of pore pressure is reduced from 8 to 3%. For Well A2, according to the logging data, the loading model is suitable for the well section from 2200 to 3500 m, and the pore pressure prediction (d = 2.5) is carried out according to the loading model in this study. The prediction results are shown in Well A2 in Fig. 13 below. The calculation results of the commonly used Eaton model are plotted in the figure. The calculation results of the prediction model in this study are compared with those of the Eaton model, except that the accuracies of the first and third pressure measuring points are similar, and the pore pressure accuracy errors are 9.6 and 4% and they decrease to 2.4 and 0.6%, respectively. Therefore, compared with the commonly used Eaton model, the comprehensive accuracy of this model is improved to 96%.

**Figure 14:**
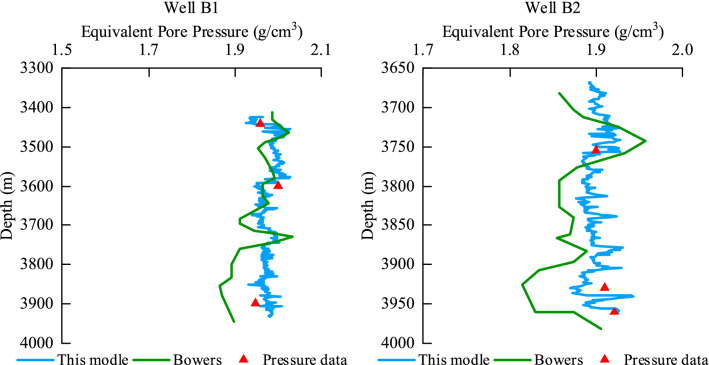
Comparison of unloading modeling prediction results.

For wells B1 and B2, according to the logging data, the unloading model is suitable for the 3400–4000 m well section and the 3650–4000 m well section. The pore pressure prediction is carried out according to the unloading model in this study. The prediction results are shown in Wells B1 and B2 in Fig. 14. The calculation results of the commonly used Bowers method model are plotted in the figure. Compared with the Bowers method model, the total accuracy error of the prediction model in this study is reduced by 2% from 9%. Therefore, compared with the commonly used Bowers method model, the comprehensive accuracy of this model is improved to 98%.

## Conclusions

1. The main pressure-forming mechanisms in the Yingqiong Basin are loading mechanism and unloading mechanism.
2. A new method of establishing a pore pressure prediction model is obtained, namely, the soil consolidation acoustic experiment method. The experimental equipment and experimental plan for simulating the loading mechanism and the unloading mechanism were designed, and the simulation method of soil consolidation and pore pressure prediction was established by means of laboratory experiments.
3. According to the new method, two pore pressure prediction models are established. The loading mechanism prediction model is shown in Eq. (5), and the unloading mechanism prediction model is shown in Eq. (8).
4. It is feasible to establish a predictive model based on soil consolidation acoustic experiments. According to the prediction model established in this study, some wells in the South China Sea Yingqiong Basin were verified and applied, and the effect of this model was evaluated. The results show that the prediction accuracy is slightly better than that of the traditional prediction model. Therefore, the soil consolidation experiment method can be used to predict pore pressure in petroleum engineering.

## Data Availability

The datasets used and analyzed during the current study available from the corresponding author on reasonable request.
